# Editorial: Advances in Analytical Features of Electrochemical Methods for the Analysis of Complicated Real Samples

**DOI:** 10.3389/fchem.2021.648920

**Published:** 2021-03-17

**Authors:** Masoud Fouladgar, Hassan Karimi-maleh, Fatih Sen

**Affiliations:** ^1^ Department of Biochemistry, Falavarjan Branch, Islamic Azad University, Isfahan, Iran; ^2^ Department of Chemical Engineering, Quchan University of Technology, Quchan, Iran; ^3^ Sen Research Group, Biochemistry Department, Faculty of Arts and Science, Dumlupinar University, Evliya Çelebi Campus, Kütahya, Turkey

**Keywords:** electrochemical methods, real sample, complicated matrices, sensors, selectivity

The aim of this topic was presentation of several features of electrochemical methods for analysis of real samples.

In the last few decades, with the technical progress of electrochemical devices, much attention has been paid to electrochemical methods for analysis of different species. However, like other analytical methods, there has been the challenge of analyzing real samples with complex matrices and several interferences.

Several techniques have been presented to improve selectivity and other figures of merit. Most of them are based on modification of electrodes and creating selective electoactive sites on the surface of electrodes (Fouladgar, 2016). In addition, the modifiers may have electrocatalytic effect on the redox of analyte.

On the other hand, application of nanomaterials has become popular because of increasing the active surface area of electrodes and consequently increasing sensitivity (Negahban et al., 2017).

Papers in this research topic, present various aspects of new methods and techniques, which have been applied for analysis of complicated samples.

As mentioned above, nanomaterials are widely used for modification of electrodes. Metal oxides are among the nanomaterials that used for this purpose. They are applied single or combination with other nanomaterials (Fouladgar and Mohammadzadeh, 2014). Naghian et al. have exhibited that using nanostructures including CdO and magnetic Fe_3_O_4_ nanoparticles can improve kinetics of the electron transfer process on the electrode. They applied this effect to determine an antiviral drug in urine, plasma and tablet. Moreover, different inherent redox potential of species or different interaction of species with electrode modifier can make simultaneous measurement possible. Accordingly, Pan et al. have reported an electrochemical method for simultaneous determination of Ascorbic acid, dopamine and uric acid in pharmaceutical samples. To reduce utilizing chemical reagents, Jing et al. have used *Plectranthus amboinicus* leaf extract for biosynthesis of gold nanoparticles. They determined nicotine in tobacco products by modifying glassy carbon with these nanoparticles.

Feng et al. presented an electrochemical method for determination of ursolic acid in a fruit extract (*Ligustrum lucidum*) which has many biological effects. Deposited composite of boron nitride nanosheets and Pt nanoparticles on the surface of glassy carbon, exhibits catalytic activity toward ursolic acid oxidation.

In addition to the analytical aspects of a sensor, biocompatibility of the sensor is important for analysis of clinical samples. Naderi Asrami et al. have prepared a biocompatible electrode containing glucose oxidase enzyme, which was covalently immobilized on a multi-layer thin film electrode. This electrode can be used to direct determination of glucose in blood serum.

Hou et al. presented another aspect of electrochemical methods based on changing piezoelectric properties of quartz crystal by deposition of analyte on it. They immobilized antibodies against B. bifidum on an Au chip and used it as an immunosensor. This kind of sensor has both sensitivity of quartz crystal and specification of immune response between antigen and antibody. Shen et al. have described application of signal amplification technique for creating an immunosensor to determine alpha-fetoprotein in serum samples. They used an immune complex consisting of alpha-fetoprotein antibody and horseradish peroxidase for immune response. In addition, the catalytic effect of a nanocomposite containing graphene oxide, methylene blue and gold nanoparticles has been applied for fabrication of this immunosensor.

One area in which application of electrochemical methods has received much attention is analysis of pharmacological and biological samples. Electrochemistry has presented simple, fast and sensitive methods to analyze these samples. Alizadeh et al. have used effects of modifying electrodes with different nanomaterials and ionic liquid.

Increasing conductivity of carbon paste subsequently increases the redox current and also increases sensitivity of the method. This technique has provided methods for determination of drugs in pharmaceutical samples (Alizadeh et al.).

Remote sensing is important for monitoring chemical species in the environment. Kim et al. have proposed a wireless sensing system for real time measuring chloride ion. They have succeeded in reducing electrolyte leakage by modifying the structure of the electrode.

Finally, the effect of species on the profile of electrochemical oscillation systems has been used for identification chemical compounds. Yan et al. have been shown that recording the oscillation profile of an oscillation reaction in the presence of different herbal medicine can be used as a fingerprint for identification of them.
